# Supplementary material for: Leveraging Existing 16SrRNA Microbial Data to Define a Composite Biomarker for Autism Spectrum Disorder
Source: Microbiol Spectr. 2022 Jun 28;10(4):e00331-22. doi: 10.1128/spectrum.00331-22 (PMC9431227; doi:10.1128/spectrum.00331-22)
Supplement: Supplemental file 1 — Supplemental material. Download spectrum.00331-22-s0001.pdf, PDF file, 0.8 MB [file spectrum.00331-22-s0001.pdf]

Supplemental legends

Table S1 Shannon diversity index, observed OTUs, and Pielou's evenness index were used to compare the differences in alpha diversity between ASD and control groups in different studies.

Table S2 Shannon diversity index, observed OTUs, and Pielou's evenness index were used to compare the differences in alpha diversity between ASD and control groups in different countries.

Table S3 Shannon diversity index, observed OTUs, and Pielou's evenness index were used to compare the differences in alpha diversity between ASD and control groups using different variable regions.

Table S4 Shannon diversity index, observed OTUs, and Pielou's evenness index were used to compare the differences in alpha diversity between ASD and control groups using different sequencing platforms.

Table S5. Comparison of fecal microbiome composition across ASD and control groups in different studies based on Bray–Curtis dissimilarity and unweighted UniFrac distance using PERMANOVA.

Table S6 Comparison of fecal microbiome composition across ASD and control groups using different variable regions based on Bray–Curtis dissimilarity and unweighted UniFrac distance using PERMANOVA

Table S7 Comparison of fecal microbiota composition groups across ASD and control groups using different sequencing platform based on Bray–Curtis dissimilarity and unweighted UniFrac distance using PERMANOVA.

Table S8 Subgroup analysis was conducted in accordance with different studies. The dominant genera in the ASD group using the LEfSe method. An LDA score (log10) of 2.0 was used as the cutoff. "Y" stands for dominant genera.

Table S9 Subgroup analysis was conducted in accordance with different studies. The dominant genera in the ASD group using the Wilcoxon rank-sum test method. "Y" stands for dominant genera.

Table S10 Subgroup analysis was conducted in accordance with different variable regions. The dominant genera in the ASD group using the LEfSe method. An LDA score

(log10) of 2.0 was used as the cutoff. “Y” stands for dominant genera.

Table S11 Subgroup analysis was conducted in accordance with different variable regions. The dominant genera in the ASD group using the LEfSe method. “Y” stands for dominant genera.

Table S12 Subgroup analysis was conducted in accordance with different sequencing platform. The dominant genera in the ASD group using the LEfSe method. An LDA score (log10) of 2.0 was used as the cutoff. “Y” stands for dominant genera.

Table S13 Subgroup analysis was conducted in accordance with different sequencing platform. The dominant genera in the control group using the LEfSe method. "Y" stands for dominant genera.

Table S14 Subgroup analysis was conducted in accordance with different studies. The dominant genera in the ASD group using the Wilcoxon rank-sum test method. “Y” stands for dominant genera.

Table S15 Subgroup analysis was conducted in accordance with different studies. The dominant genera in the control group using the Wilcoxon rank-sum test method. “Y” stands for dominant genera.

Table S16 Subgroup analysis was conducted in accordance with different variable regions. The dominant genera in the ASD group using the Wilcoxon rank-sum test method. “Y” stands for dominant genera.

Table S17 Subgroup analysis was conducted in accordance with different variable regions. The dominant genera in the control group using the Wilcoxon rank-sum test method. “Y” stands for dominant genera.

Table S18 Subgroup analysis was conducted in accordance with different sequencing platform. The dominant genera in the ASD group using the Wilcoxon rank-sum test method. “Y” stands for dominant genera.

Table S19 Subgroup analysis was conducted in accordance with different sequencing platform. The dominant genera in the control group using the Wilcoxon rank-sum test method. “Y” stands for dominant genera.

Table S1

project	Shannon diversity index	observed OTUs	Pielou's evenness index
S1	0.631	0.423	0.522
S2	0.148	0.128	0.291
S3	0.606	0.449	0.812
S4	0.715	0.916	0.749
S5	< 0.001*	< 0.001*	< 0.001*
S6	0.497	0.109	0.640
S7	0.350	0.222	0.604
S8	0.009*	0.009*	0.027*
S9	0.030*	< 0.001*	0.129
S10	0.038*	0.033*	0.112

P < 0.05*

Table S2

Country	Study included	Shannon diversity index	Observed OTUs	Pielou's evenness index
China	S2, S4, S5, S6, S8, S9	0.640	0.865	0.696
Ecuador	S7	0.369	0.245	0.639
Italy	S1, S10	0.211	0.211	0.170
Korean	S3	0.590	0.539	0.763

P < 0.05*

Table S3

Sequencing Region	Study included	Shannon diversity index	Observed OTUs	Pielou's evenness index
V3-4	S1, S2, S3, S5, S8, S10	0.082	0.036*	0.120
V4	S6, S7, S9	0.570	0.331	0.670
V4-5	S4	0.841	0.844	0.668

P < 0.05*

Table S4

Sequencing platform	Study included	Shannon diversity index	Observed OTUs	Pielou's evenness index
Illumina HiSeq 2500	S6	0.518	0.096	0.683
Illumina HiSeq 4000	S9	0.314	< 0.001*	0.115
Illumina MiSeq	S1, S2, S3, S4, S5, S7, S8, S10	0.980	0.702	0.893

$P < 0.05^*$

Table S5

Study	Bray_Curtis		unweighted_unifrac	
	Pseudo-F	p-value	Pseudo-F	p-value
S1	0.775	0.815	0.992	0.372
S2	1.747	0.007*	2.122	0.002*
S3	1.260	0.127	1.291	0.100
S4	2.101	0.005*	0.966	0.451
S5	15.550	0.001*	10.700	0.001*
S6	6.260	0.001*	8.282	0.001*
S7	1.030	0.370	0.977	0.457
S8	1.316	0.056	1.293	0.066
S9	3.746	0.001*	3.239	0.001*
S10	3.130	0.001*	2.100	0.003*

PERMANOVA, Permutational multivariate analysis of variance;
$P < 0.05^*$

Table S6

Sequencing Region	Study included	Bray_Curtis		unweighted_unifrac	
		Pseudo-F	p-value	Pseudo-F	p-value
V3-4	S1, S2, S3, S5, S8, S10	2.858	0.001*	3.194	0001*
V4	S6, S7, S9	2.066	0.005*	1.706	0.053
V4-5	S4	4.736	0.001*	5.224	0001*

PERMANOVA, Permutational multivariate analysis of variance;
$P < 0.05^*$

Table S7

Country	Study included	Bray_Curtis		unweighted_unifrac	
		Pseudo-F	p-value	Pseudo-F	p-value
Illumina HiSeq 2500	S6	6.224	0.001*	8.335	0.001*
Illumina HiSeq 4000	S9	3.805	0.001*	3.984	0.001*
Illumina MiSeq	S1, S2, S3, S4, S5, S7, S8, S10	3.014	0.001*	3.697	0.001*

PERMANOVA, Permutational multivariate analysis of variance;
$P < 0.05^*$

able S8

**Genera**	S1	S2	S3	S4	S5	S6	S7	S8	S9	S10
Alistipes									Y	
Anaerostipes					Y	Y			Y	
Bacteroides					Y					Y
Bifidobacterium			Y		Y	Y				
Blautia					Y	Y				
Clostridium_IV						Y				
Clostridium_sensu_stricto									Y	
Clostridium_XlVa					Y					
Clostridium_XVIII					Y				Y	
Collinsella		Y	Y			Y			Y	
Coprococcus					Y				Y	
Dialister						Y				
Dorea				Y					Y	
Enterococcus									Y	
Escherichia_Shigella				Y		Y				
Eubacterium						Y				
Faecalibacterium										Y
Fusicatenibacter					Y					
Gemmiger						Y				
Lachnospiracea_incertae_sedis			Y						Y	
Megamonas					Y			Y		
Megasphaera						Y				
Morganella				Y						
Oscillibacter							Y			
Parabacteroides					Y					
Parasutterella					Y	Y				
Prevotella					Y			Y	Y	
Romboutsia					Y				Y	
Ruminococcus2					Y				Y	
Streptococcus					Y					

Table S9

Genera	S1	S2	S3	S4	S5	S6	S7	S8	S9	S10
Akkermansia					Y					
Anaerostipes										Y
Bacteroides			Y						Y	
Barnesiella	Y									
Bifidobacterium										Y
Blautia								Y		Y
Clostridium_XlVa									Y	
Collinsella										Y
Dialister					Y					
Escherichia_Shigella					Y					
Eubacterium										Y
Faecalibacterium									Y	
Fusicatenibacter								Y		
Gemmiger		Y								
Haemophilus					Y					
Intestinibacter								Y		
Lactobacillus							Y			
Megamonas						Y				
Parabacteroides				Y		Y				
Parasutterella									Y	
Phascolarctobacterium		Y				Y				
Prevotella		Y				Y				
Romboutsia								Y		
Roseburia				Y	Y					
Ruminococcus2						Y				Y
Streptococcus								Y		Y
Veillonella									Y	

Table S10

Genera	S1	S2	S3	S4	S5	S6	S7	S8	S9	S10
Acidaminococcus						Y				
Aggregatibacter										Y
Alistipes										Y
Allisonella			Y							
Anaerofilum									Y	
Anaerostipes	Y					Y				
Anaerotruncus		Y								
Bacteroides							Y			
Bifidobacterium						Y				
Bilophila						Y				
Blautia						Y				
Bradyrhizobium				Y						
Butyrivibrio					Y					
Christensenella						Y				
Chryseobacterium						Y				
Clostridium_IV									Y	
Clostridium_sensu_stricto									Y	
Clostridium_XI			Y	Y	Y					
Clostridium_XlVa					Y		Y			Y
Clostridium_XlVb					Y					
Clostridium_XVIII					Y				Y	
Collinsella		Y	Y			Y			Y	
Comamonas						Y				
Coprobacter									Y	
Coprococcus									Y	
Desulfovibrio				Y					Y	
Dialister						Y			Y	
Dorea									Y	
Eisenbergiella						Y				
Enterococcus		Y		Y						
Escherichia/Shigella				Y		Y				
Eubacterium						Y				
Faecalibacterium					Y					Y

Fusobacterium								Y		
Gemmiger					Y					
Holdemania							Y			
Lachnoanaerobaculum						Y				
Lachnospira			Y		Y					
Lachnospiracea_incertae_sedis			Y		Y				Y	
Lactobacillus			Y							
Leuconostoc									Y	
Megasphaera										Y
Mitsuokella						Y				
Odoribacter					Y					
Oscillibacter										Y
Pandoraea				Y						
Paracoccus						Y				
Paraeggerthella									Y	
Parasutterella									Y	
Parvimonas				Y						
Pectobacterium						Y				
Phascolarctobacterium									Y	
Phyllobacterium				Y						
Prevotella				Y				Y		
Pseudobutyrivibrio					Y					
Pseudomonas					Y			Y		
Roseburia					Y					
Rothia						Y				
Ruminococcus						Y				
Senegalimassilia									Y	
Streptococcus									Y	
Vibrio						Y				
Weissella					Y					

Table S11

Genera	S1	S2	S3	S4	S5	S6	S7	S8	S9	S10
Abiotrophia					Y					Y
Acetanaerobacterium		Y								
Acidaminococcus					Y					
Actinomyces										Y
Aggregatibacter					Y					
Akkermansia					Y					
Alcaligenes				Y						
Alistipes						Y				
Alloprevotella						Y			Y	
Anaerofustis					Y					Y
Anaeroglobus						Y				
Anaerostipes				Y	Y					
Arthrobacter									Y	
Atopobium										Y
Bacteroides			Y			Y			Y	
Barnesiella					Y					
Bifidobacterium					Y					Y
Bilophila		Y								
Blautia										Y
Buttiauxella			Y		Y					
Butyricicoccus						Y				
Butyricimonas							Y			
Campylobacter					Y					
Catabacter		Y			Y					
Cellulosilyticum						Y				
Christensenella		Y			Y					
Citrobacter			Y							
Clostridium_III						Y				
Clostridium_IV					Y					
Clostridium_XlVa						Y			Y	

Clostridium_XlVb						Y			Y	
Collinsella					Y					Y
Comamonas		Y					Y			
Coprobacter					Y					
Corynebacterium										Y
Desulfovibrio					Y	Y		Y		
Dialister					Y					
Dorea		Y			Y					
Eggerthella										Y
Enterobacter					Y					
Enterococcus									Y	Y
Erysipelotrichaceae_incertae_sedis					Y					
Escherichia/Shigella					Y					
Eubacterium				Y	Y					
Ezakiella					Y					
Faecalicoccus						Y				
Flavonifractor					Y					
Fusobacterium					Y					
Gemella					Y					Y
Gemmiger		Y								
Haemophilus			Y		Y					
Halomonas						Y				
Howardella				Y						
Intestinibacter			Y		Y					
Intestinimonas					Y	Y			Y	
Johnsonella									Y	
Klebsiella			Y							
Lachnoanaerobaculum					Y					
Lactobacillus						Y				
Lactonifactor					Y					
Megamonas						Y				
Megasphaera					Y					
Odoribacter						Y				
Olsenella									Y	
Oscillibacter					Y					
Pantoea					Y					
Parabacteroide						Y			Y	

s										
Paraprevotella						Y				
Parasutterella						Y				
Parvimonas					Y					
Peptoniphilus		Y								
Peptostreptoc occus					Y					
Phascolarctob acterium					Y			Y		
Porphyromon as					Y					
Prevotella		Y				Y				
Pseudobutyriv ibrio								Y		
Pseudoflavoni fractor						Y				
Pseudomonas										Y
Raoultella					Y					
Romboutsia					Y					
Roseburia				Y				Y		
Rothia					Y					Y
Ruminococcu s					Y					
Ruminococcu s2					Y					
Saccharoferm entans		Y								
Salmonella		Y			Y					
Sphingobacter ium									Y	
Stenotrophom onas						Y				
Streptococcus					Y					Y
Sutterella						Y				
Turicibacter					Y	Y				
Veillonella					Y					

Table S12

**Genera**	**V3-4**	**V4**	**V4-5**
Anaerostipes		Y	
Bacteroides	Y		
Bifidobacterium		Y	
Blautia		Y	
Collinsella		Y	
Dorea		Y	Y
Escherichia_Shigella		Y	Y
Eubacterium		Y	
Gemmiger		Y	
Intestinibacter		Y	
Lachnospiracea_incertae_sedis	Y		
Megamonas	Y		
Morganella			Y
Parasutterella	Y		
Romboutsia		Y	
Streptococcus		Y	

Table S13

Genera	V3-4	V4	V4-5
Akkermansia	Y		
Bacteroides		Y	
Clostridium_XVIII		Y	
Dialister	Y		
Gemmiger	Y		
Megamonas		Y	
Parabacteroides		Y	Y
Phascolarctobacterium		Y	
Prevotella		Y	
Roseburia			Y

Table S14

Genera	V3-4	V4	V4-5
Alistipes		Y	
Alloprevotella		Y	
Anaeroglobus		Y	
Arthrobacter		Y	
Atopobium	Y		
Bacteroides	Y	Y	
Brachymonas		Y	
Bradyrhizobium			Y
Butyricicoccus		Y	
Butyricimonas		Y	
Butyrivibrio	Y		
Cellulosilyticum		Y	
Clostridium_III		Y	
Clostridium_XI			Y
Clostridium_XlVa	Y	Y	
Clostridium_XlVb	Y	Y	
Clostridium_XVIII		Y	
Collinsella	Y		
Comamonas		Y	
Desulfovibrio		Y	Y
Enterococcus			Y
Escherichia/Shigella			Y
Eubacterium		Y	
Faecalicoccus		Y	
Halomonas	Y	Y	
Intestinimonas		Y	
Lachnospira	Y		
Lachnospiracea_incertae_sedis	Y		
Megamonas		Y	
Odoribacter		Y	
Olsenella		Y	
Oribacterium		Y	
Pandoraea			Y
Parabacteroides		Y	
Paraprevotella		Y	
Parvimonas			Y
Phyllobacterium			Y
Prevotella		Y	Y
Pseudoflavonifractor		Y	
Stenotrophomonas		Y	

Sutterella		Y	

Table S15

Genera	V3-4	V4	V4-5
Akkermansia	Y		
Alcaligenes			Y
Alistipes		Y	
Alloprevotella		Y	
Anaerofustis	Y		
Anaeroglobus		Y	
Anaerostipes			Y
Bacteroides		Y	
Blautia	Y		
Brachymonas		Y	
Bradyrhizobium	Y		
Butyricicoccus		Y	
Butyricimonas		Y	
Catabacter	Y		
Cellulosilyticum		Y	
Christensenella	Y		
Clostridium_III		Y	
Clostridium_IV	Y		
Clostridium_sensu_stricto	Y		
Clostridium_XlVa		Y	
Clostridium_XlVb		Y	
Clostridium_XVIII		Y	
Comamonas		Y	
Desulfovibrio	Y	Y	
Dialister	Y		
Dorea	Y		
Erysipelotrichaceae_incertae_sedis	Y		
Eubacterium	Y	Y	Y
Ezakiella	Y		
Faecalicoccus		Y	
Flavonifractor	Y		
Granulicatella	Y		
Haemophilus	Y		
Halomonas		Y	
Howardella			Y
Intestinibacter	Y		
Intestinimonas		Y	
Lactonifactor	Y		
Megamonas		Y	
Odoribacter		Y	

Oribacterium		Y	
Pantoea	Y		
Parabacteroides		Y	
Paraprevotella		Y	
Parvimonas	Y		
Peptoniphilus	Y		
Phascolarctobacterium	Y		
Prevotella		Y	
Pseudoflavonifractor		Y	
Romboutsia	Y		
Roseburia			Y
Ruminococcus	Y		
Ruminococcus2	Y		
Stenotrophomonas		Y	
Streptococcus	Y		
Sutterella		Y	
Turicibacter	Y		
Veillonella	Y		

Table S16

**Genera**	**Illumina MiSeq**	**Illumina HiSeq 2500**	**Illumina HiSeq 4000**
Alistipes			Y
Anaerostipes		Y	Y
Bacteroides	Y		
Bifidobacterium		Y	
Blautia		Y	
Clostridium_IV		Y	
Clostridium_sensu_stricto			Y
Clostridium_XVIII			Y
Collinsella	Y	Y	Y
Coprococcus			Y
Dialister		Y	
Dorea			Y
Enterococcus			Y
Escherichia_Shigella		Y	
Eubacterium		Y	
Gemmiger		Y	
Lachnospiracea_incertae_sedis	Y		Y
Megamonas	Y		
Megasphaera		Y	
Parasutterella		Y	
Prevotella			Y
Romboutsia			Y
Ruminococcus2	Y		Y
Subdoligranulum			Y

Table S17

**Genera**	**Illumina MiSeq**	**Illumina HiSeq 2500**	**Illumina HiSeq 4000**
Bacteroides			Y
Clostridium_XlVa			Y
Clostridium_XVIII	Y		
Dialister	Y		
Faecalibacterium			Y
Intestinibacter	Y		
Megamonas		Y	
Parabacteroides		Y	
Parasutterella			Y
Phascolarctobacterium		Y	
Prevotella		Y	
Ruminococcus	Y		
Ruminococcus2		Y	
Veillonella			Y

Table S18

Genera	Illumina MiSeq	Illumina HiSeq 2500	Illumina HiSeq 4000
Acidaminococcus		Y	
Anaerofilum			Y
Anaerostipes		Y	
Bacteroides	Y		
Bifidobacterium		Y	
Bilophila		Y	
Blautia		Y	
Butyrivibrio	Y		
Christensenella		Y	
Chryseobacterium		Y	
Clostridium_IV			Y
Clostridium_sensu_stricto			Y
Clostridium_XlVb	Y		
Clostridium_XVIII			Y
Collinsella	Y	Y	Y
Comamonas		Y	
Coprobacter			Y
Coprococcus			Y
Desulfovibrio			Y
Dialister		Y	Y
Dorea			Y
Eisenbergiella		Y	
Escherichia/Shigella		Y	
Eubacterium		Y	
Faecalibacterium	Y		
Halomonas	Y		
Lachnoanaerobaculum		Y	
Lachnospira	Y		
Lachnospiracea_incertae_sedis			Y
Leuconostoc			Y
Mitsuokella		Y	
Odoribacter	Y		
Pandoraea	Y		
Paracoccus		Y	
Paraeggerthella			Y
Parasutterella			Y
Pectobacterium		Y	
Phascolarctobacterium			Y
Pseudobutyrivibrio	Y		

Pseudomonas	Y		
Rothia		Y	
Ruminococcus		Y	
Senegalimassilia			Y
Streptococcus	Y		Y
Veillonella	Y		
Vibrio		Y	

Table S19

Genera	Illumina MiSeq	Illumina HiSeq 2500	Illumina HiSeq 4000
Acinetobacter	Y		
Alcaligenes	Y		
Alistipes		Y	
Alloprevotella		Y	Y
Anaeroglobus		Y	
Anaerostipes	Y		
Arthrobacter			Y
Bacteroides		Y	Y
Blautia	Y		
Butyricicoccus		Y	
Catabacter	Y		
Cellulosilyticum		Y	
Christensenella	Y		
Clostridium_III		Y	
Clostridium_sensu_stricto	Y		
Clostridium_XI	Y		
Clostridium_XlVa		Y	Y
Clostridium_XlVb		Y	Y
Clostridium_XVIII	Y		
Desulfovibrio		Y	
Dialister	Y		
Dorea	Y		
Eisenbergiella	Y		
Enterococcus			Y
Erysipelotrichaceae_incertae_sedis	Y		
Eubacterium	Y		
Ezakiella	Y		
Faecalicoccus		Y	
Haemophilus	Y		
Halomonas		Y	
Intestinibacter	Y		
Intestinimonas	Y	Y	Y
Johnsonella			Y
Lactobacillus		Y	
Lactonifactor	Y		
Megamonas		Y	
Odoribacter		Y	
Olsenella			Y
Parabacteroides		Y	Y

Paraprevotella		Y	
Parasutterella		Y	
Porphyromonas	Y		
Prevotella		Y	
Pseudoflavonifractor		Y	
Romboutsia	Y		
Ruminococcus2	Y		
Sphingobacterium			Y
Stenotrophomonas			Y
Sutterella		Y	
Turicibacter		Y	
